# Two-dimensional speckle tracking echocardiography predicts early subclinical cardiotoxicity associated with anthracycline-trastuzumab chemotherapy in patients with breast cancer

**DOI:** 10.1186/s12885-018-4935-z

**Published:** 2018-10-25

**Authors:** Maria C. Arciniegas Calle, Nicole P. Sandhu, Hongmei Xia, Stephen S. Cha, Patricia A. Pellikka, Zi Ye, Joerg Herrmann, Hector R. Villarraga

**Affiliations:** 1 Department of Cardiovascular Medicine, Mayo Clinic, Rochester, MN USA; 2 Division of General Internal Medicine, Mayo Clinic, Rochester, MN USA; 3 Division of Biostatistics, Mayo Clinic, Scottsdale, AZ USA; 4 Department of Cardiovascular Diseases, Mayo Clinic, 200 First St SW, Rochester, MN 55905 USA

**Keywords:** Breast neoplasms, Cardiotoxicity, Chemotherapy, Heart failure

## Abstract

**Background:**

Combined anthracycline-trastuzumab chemotherapy has been associated with LV dysfunction. We aimed to assess early changes in left ventricular (LV) and right ventricular (RV) mechanics associated with combined anthracycline-trastuzumab treatment for breast cancer. As well as explore whether early changes in 2-dimensional (2D)–speckle tracking echocardiography (STE) could predict later chemotherapy-induced cardiotoxicity.

**Methods:**

Sixty-six patients with breast cancer who received anthracycline-trastuzumab treatment were included (mean [±SD] age, 52 [9] years). Echocardiograms were available for analysis with 2D-STE at the following time points: pretreatment (T0), first cycle (T1), and second cycle (T2) of combined chemotherapy. All patients had a normal pretreatment LV ejection fraction (LVEF). Cardiotoxicity was defined as a decrease in LVEF of at least 10 percentage points from baseline on follow-up echocardiography.

**Results:**

Cardiotoxicity developed in 13 of the 66 patients (20%). The mean (±SD) LVEF at T0 was 66% (±6); at T1 60% (±7); and at T2, 54% (±6). For the 53 patients without cardiotoxicity, the LVEF was 65% (±4%) at T0, 63% (±5%) at T1, and 62% (±4) at T2. Global longitudinal strain (GLS) at T1 was the strongest indicator of subsequent cardiotoxicity (area under the curve, 0.85; cutoff value, − 14.06; sensitivity, 91%; specificity, 83%; *P* = .003). Compared with baseline (T0), left ventricular longitudinal strain, LV circumferential strain, circumferential peak systolic strain rate (SR), circumferential peak early diastolic SR, right ventricular longitudinal strain, and longitudinal peak systolic SR at T1 and T2 were reduced significantly in patients with cardiotoxicity (*P* < .05).

**Conclusions:**

Anthracycline-trastuzumab treatment leads to early deterioration of LV GLS, circumferential strain, and systolic SR. Right ventricular GLS and SR were also affected. Early changes in GLS are good predictors of subsequent development of anthracycline-trastuzumab–induced cardiotoxicity.

## Background

Cardiotoxicity due to combined chemotherapy is a leading cause of morbidity and mortality for survivors of breast cancer [1–4], and the survival rate for patients who subsequently develop heart failure is as low as 25% at 5 years [5]. Early cardiotoxicity may be *silent*, yet its prompt diagnosis is important for patients with early structural heart changes but no signs or symptoms of heart failure (stage B heart failure [American Heart Association/American College of Cardiology]) [6].

Combined chemotherapeutic agents, such as anthracycline and trastuzumab have increased survival rates for patients with HER 2 positive breast cancer, and combination therapy has thus become a well-established therapeutic approach [7]. However, anthracyclines may generate dose-dependent left ventricular (LV) dysfunction, which is associated with poor prognosis [8]. In addition, trastuzumab results in cardiac dysfunction in 3% to 5% of treated patients [7, 8]. Potential cardiotoxicity of therapy, focused on early detection of minor LV myocardial dysfunction and early intervention, should be considered for patients undergoing cancer therapy with these agents [1, 6, 9]. Early recognition and appropriate therapy can improve outcomes and decrease morbidity, mortality, and progression to clinical heart failure [10].

Monitoring for early signs of cardiotoxicity during a patient’s treatment with chemotherapy can be done by using strain measurements [11]. Two-dimensional–speckle tracking echocardiography (2D-STE) is a promising technique that can evaluate cardiac mechanics in the 3 domains of contractility [12–16]. A growing body of literature supports the use of myocardial deformation parameters to detect early myocardial injury (stage B heart failure) and to forecast ventricular dysfunction in patients receiving cancer therapy [16–23]. Because very limited data exist about the early cardiotoxicity of chemotherapy in patients taking both anthracycline and trastuzumab [22] or about right ventricular (RV) mechanics in patients who receive cancer therapy, we designed a study aimed to detect early changes in LV and RV mechanics and to determine if 2D-STE could predict preclinical cardiotoxicity from anthracycline-trastuzumab treatment after the first and second cycle of chemotherapy in patients with breast cancer.

## Methods

### Study population

For this retrospective study, we enrolled women newly diagnosed with breast cancer between December 1, 2004, and June 1, 2012, who were treated with an anthracycline (doxorubicin and epirubicin), or a HER 2 inhibitor (trastuzumab), or both. Though the majority of the study population (40/66; 61%) received adjuvant therapy, a substantial number (26/66; 39%); received neoadjuvant therapy. Of the total cohort, one woman was found to have stage IV disease at diagnosis. Twenty three (35%) had stage III disease and 6 of those had inflammatory breast cancer. None of the study population received concurrent anthracyclines and trastuzumab. The average age was 52 (±9), mean anthracycline dose of 252 (±45) mg/m^2^ and trastuzumab was administered for an average of 11 (±2.7) months in 3 week cycles and a mean dose of 5.4 (±2.7) gr. All patients had to have a normal pretreatment (T0) LV ejection fraction (LVEF), had to have been followed up for 1 year, as well as an echocardiogram at baseline. Patients who met the inclusion criteria and had images of adequate quality, as well as follow-up, were included (*N* = 66). Because of lack of consensus, we followed the definition of cardiotoxicity used in the clinical trials for combined chemotherapy in HER 2 positive patients, where it was defined as an absolute decrease in LVEF of 10 or more percentage points from baseline echocardiogram [24, 25]. Traditional cardiovascular risk factors, such as age, hypertension, diabetes mellitus, hyperlipidemia, family history of premature coronary artery disease, and smoking status were also considered in the analyses [26–28]. The study was approved by the Mayo Clinic Institutional Review Board, and written informed consent was obtained.

### Imaging acquisition and speckle tracking analysis

To be included, patients needed to have at least 3 standard echocardiographic examinations: at baseline (T0); from the start of chemotherapy to first echocardiogram (T1) and from the start of chemotherapy to the second echocardiogram (T2) following the cardio oncology clinic protocol [19]. Echocardiographic examinations were performed using a GE Vivid 7 system (General Electric Company) with an M4S transducer (1.5-4.3 MHz). Mean frame rates were 55 Hz for grayscale imaging. Studies were performed and reported according to the guidelines of the American Society of Echocardiography [29]. LVEF was calculated using the biplane Simpson method [13]. Digital images were saved for subsequent, blinded off-line analysis using the Syngo Velocity Vector Imaging software, version 3.5 and 2D-STE analyses were performed.

For all patients, the region of interest analyzed was adjusted to cover at least 90% of the myocardial wall thickness for myocardial strain and SR. LV longitudinal parameters were measured from the apical 4-chamber, 2-chamber, and 3-chamber views, and the myocardium was divided into 6 segments per view. Care was taken to ensure that the long axis of the ventricle was perpendicular to the plane of the mitral annulus in the LV apical views. Circumferential and radial parameters were measured using the parasternal short-axis plane at the midventricular level. RV longitudinal parameters were measured from the apical 4-chamber view. Global and segmental myocardial deformation parameters, including strain (S), peak systolic strain rate (SRs), and peak early diastolic strain rate (SRe) were measured for each patient.

### Reproducibility

To determine intraobserver variability, echocardiograms from 20 randomly assigned patients were reanalyzed by the same observer (X.H.) 2 months after the initial analysis. For interobserver variability, the same patients and the same cardiac cycles were analyzed by a second observer (Z.Y.).

### Statistical analysis

Continuous data are presented as mean (SD) and categorical data as frequencies (percentages). Deformation parameters were compared among T0, T1, and T2 using 1-way ANOVA and paired *t* tests. Differences among age subgroups were assessed using the Tukey-Kramer multiple comparisons test. The first(T1) and second time point(T2) were used to construct a receiver operating characteristic (ROC) curve, which was used to predict cardiotoxicity. The best cutoff value was defined as the point with the highest sum of sensitivity and specificity. Univariate and multivariate logistic regression analyses were used to determine predictors of a significant decrease in LVEF. Intraobserver variability, interobserver variability, and intraclass correlation coefficients (ICCs) with 95% CIs were calculated to evaluate test reliability [30, 31]. All statistical analyses were performed using SAS software, version 9.3 (SAS Institute Inc). A *P* value <.05 was considered statistically significant.

## Results

### Patient characteristics

Sixty-six patients who completed anthracycline-trastuzumab treatment for breast cancer were included in the study (mean [±SD] age, 52 [±9] years; median [range] age, 51 [34-72] years). The patients’ clinical characteristics are summarized in Table 1. Of the 66 patients, 13 (20%) had cardiotoxicity defined by a decrease in LVEF of 10 or more percentage points from baseline [24, 25]. Forty-six percent of those patients developed cardiotoxicity at T1 and 54% at T2. Both groups were followed up for the same amount of time. The median cumulative doses of anthracycline-trastuzumab are shown in Table 1. The patients LVEF in whom cardiotoxicity developed vs. patients LVEF with no cardiotoxicity is shown in Table 2. Thirty-one patients had baseline cardiac risk factors, including hypertension, 13 patients; diabetes mellitus, 3 patients; hyperlipidemia, 12 patients; family history of premature coronary artery disease, 4 patients; and smoking history, 13 patients. There was no difference in cardiotoxicity for patients with more than or less than 3 risk factors. In addition, 50 patients (76%) began radiotherapy 5.3 (2.2) months after the start of chemotherapy.

**Table 1:** Characteristics of the Study Population

Characteristic	Patients, No. (SD/%)^a,b^ *N* = 66
Clinical characteristics	
Age, mean (SD), y	52 (9)
Dose, mg/m^2^, median^c^	
T1: anthracycline, 440	54 (82)
T1: trastuzumab, 1560	31 (47)
T2: anthracycline, 445	66 (100)
T2: trastuzumab, 2440	66 (100)
Risk factors	
Cardiovascular risk factors	31 (47)
Hypertension	13 (20)
Diabetes mellitus	3 (5)
Hyperlipidemia	12 (18)
Family history of premature CAD	4 (6)
Smoking history	13 (20)
Radiotherapy	50 (76)
Heart rate, baseline echocardiogram, mean (SD), bpm	71 (12)
Cardiotoxicity (a decrease ≥10% to EF ≤53%)	13 (20)

*Abbreviations*: *bpm* beats per minute, *CAD* coronary artery disease, *EF* ejection fraction, *T0* baseline (pretreatment), *T1* time from start of chemotherapy to first echocardiogram (median, 2.85 months), *T2* time from start of chemotherapy to second echocardiogram (median, 5.44 [4.61-6.47] months)

^a^Unless otherwise indicated

^b^Data are expressed as value (%) for categorical data and mean (SD) for continuous data

^c^T1: 2.25 (median) months from the start of chemotherapy to the first echocardiogram; T2: 5.44 (4.61-6.47) months from the start of chemotherapy to the second echocardiogram

**Table 2:** LVEF in Patients With and Without Cardiotoxicity for Time Points T0, T1, and T2

Time	LVEF, %^a^	
	Patients With Cardiotoxicity (*n* = 13)	Patients Without Cardiotoxicity (*n* = 53)
T0	66 (±6)	65 (±4)
T1	60 (±7)	63 (±5)
T2	54 (±6)	63 (±4)

*Abbreviations*: *LVEF* left ventricular ejection fraction, *T0* baseline (pretreatment), *T1* time from start of chemotherapy to first echocardiogram, *T2* time from start of chemotherapy to second echocardiogram

^a^Data are expressed as mean (±SD)

### LV and RV mechanics at T0, T1, and T2

Echocardiographic follow up was obtained in three time points: baseline (T0) at the time of diagnosis; (T1) from the start of chemotherapy to first echocardiogram (median [interquartile range {IQR}], 2.25 [1.84-2.99] months); and (T2) from the start of chemotherapy to the second echocardiogram (T2) (median [IQR], 5.44 [4.61-6.47] months).

**Fig. 1:**
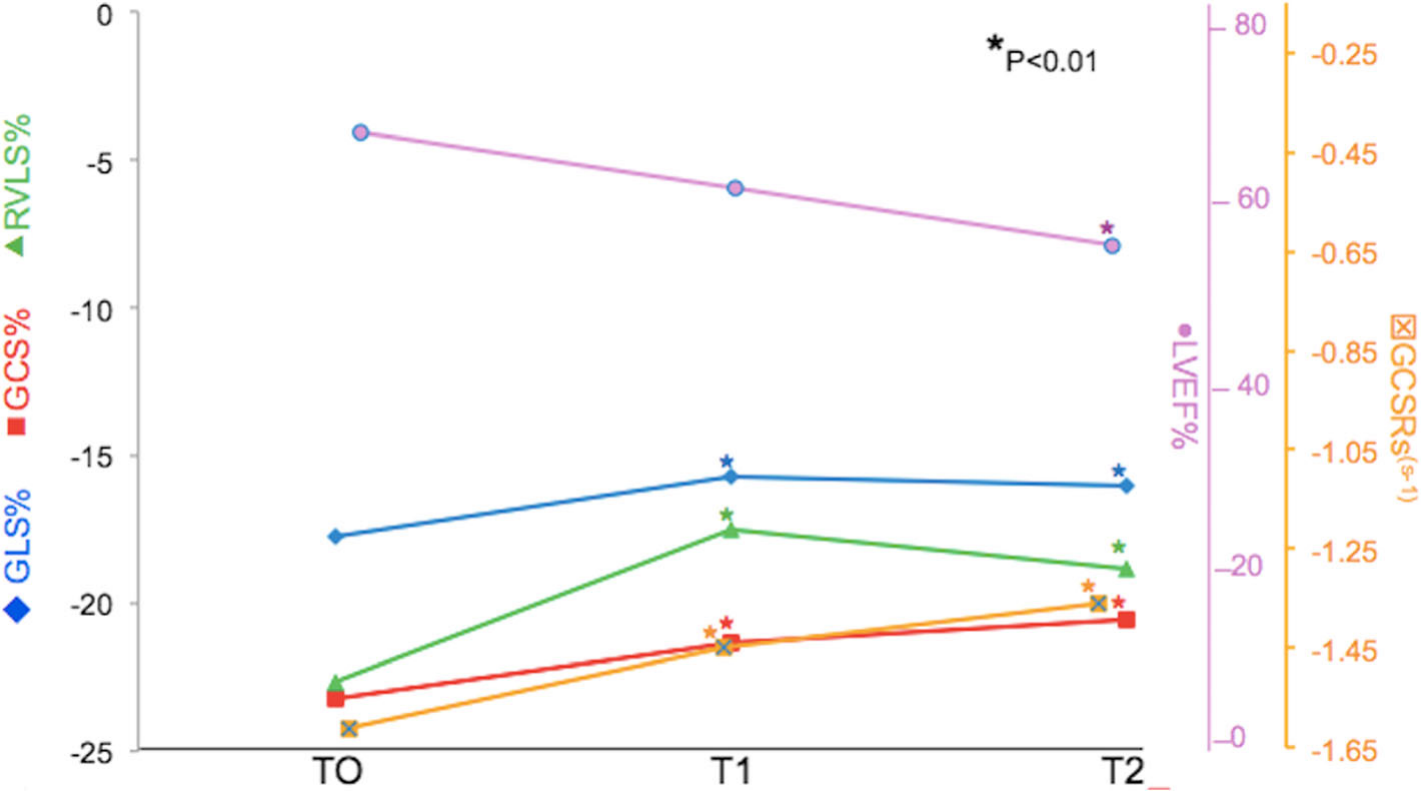
Dynamic changes of LVEF, GLS, GCS, RVLS and GLSRs at different time points (T0-T2). Multi-line graph showing the relationship between LVEF% (purple line), GLS% (blue line), RVLS%(green line), GCS% (red line) and GCSRs (orange line), at three different time points: T0, T1, T2. Notice that there is significant change between T0 and T1 for right ventricular longitudinal strain (RVLS), global circumferential strain (GCS), global longitudinal strain (GLS), and global circumferential systolic strain rate (GCSRs) at T1 that can predict the development of cardiac toxicity at T2

Serial 2D-STE parameters at T0, T1, and T2 are summarized in Fig. 1. Compared with T0, GLS at T1 and T2 and GCS at T1 and T2 were significantly reduced (*P* < .01 for all). Also significantly reduced were RV GLS at T1, SRs at T1, RV GLS at T2, SRs at T2 and SRe at T1 (*P* < .001 for all). There was a significant decrease with increasing age in LV GLS both at T1 and T2, in LV longitudinal SRe at T2, and in RV longitudinal SRe at T1 (*P* < .01 for all). There was no significant decrease in any parameter measured with increasing age (Table 3).

**Table 3:** LV and RV Functional Parameters Compared by Patient Age for T0, T1, and T2^a^

Parameter	T0				T1				T2			
	< 50 y (*n* = 30)	50-59 y (*n* = 25)	≥60 y (*n* = 11)	*P* Value	< 50 y (*n* = 30)	50-59 y (*n* = 25)	≥60 y (*n* = 11)	*P* Value	< 50 y (*n* = 30)	50-59 y (*n* = 25)	≥60 y (*n* = 11)	*P* Value
EF, %	64.16 (4.14)	65.56 (4.65)	66.50 (4.75)	.17	61.53 (4.51)	61.70 (5.68)	61.38 (10.93)	.99	59.81 (3.73)	60.19 (6.44)	60.80 (5.54)	.052
LV longitudinal												
S, %	−18.34 (2.85)	−17.69 (2.86)	− 17.31 (2.34)	.39	−16.82 (2.74)	− 14.35 (2.79)^b^	− 14.77 (2.53)^b^	.007	− 17.03 (3.27)	− 15.33 (2.71)^b^	−15.06 (2.79)^b^	.009
SRs, l/s	−1.05 (0.17)	−1.08 (0.18)	− 1.03 (0.15)	.58	− 1.09 (0.19)	−0.97 (0.22)	−1.00 (0.25)	.12	−1.03 (0.16)	−0.99 (0.18)	− 0.92 (0.19)	.22
SRe, l/s	1.16 (0.29)	1.06 (0.26)	1.02 (0.22)	.14	1.11 (0.25)	1.00 (0.28)	1.04 (0.37)	.35	1.13 (0.29)	1.00 (0.22)	0.84 (0.26)^b^	.009
LV circumferential												
S, %	−23.02 (3.48)	−22.81 (4.58)	−24.13 (5.21)	.56	−21.52 (3.76)	−20.47 (3.53)	− 22.02 (4.47)	.40	− 20.21 (4.31)	−19.71 (4.88)	− 23.26 (3.66)	.08
SRs, l/s	−1.51 (0.28)	−1.73 (0.36)	− 1.61 (0.52)	.11	− 1.42 (0.26)	−1.36 (0.30)	− 1.41 (0.38)	.05	− 1.32 (0.32)	−1.34 (0.35)	− 1.54 **(**0.45)	.20
SRe, l/s	1.57 (0.34)	1.75 (0.54)	1.50 (0.51)	.18	1.46 (0.40)	1.49 (0.35)	1.49 (0.47)	.96	1.34 (0.32)	1.39 (0.43)	1.53 (0.56)	.43
LV radial												
S, %	41.52 (12.58)	35.09 (13.13)	38.83 (11.18)	.16	37.73 (13.92)	38.65 (10.35)	37.32 (13.53)	.90	38.13 (13.09)	35.58 (12.55)	35.91 (11.51)	.74
SRs, l/s	2.41 (0.82)	2.82 (2.67)	2.29 (0.76)	.52	2.08 (0.62)	2.33 (0.63)	2.24 (0.78)	.38	2.03 (0.69)	2.15 (0.95)	1.82 (0.64)	.51
SRe, l/s	−2.53 (1.12)	−2.31 (0.85)	−2.14 (1.05)	.41	− 2.10 (0.69)	− 2.41 (0.99)	−2.24 (1.00)	.45	−2.18 (0.93)	− 2.25 (1.12)	−1.92 (0.95)	.66
RV longitudinal												
S, %	−23.95 (7.82)	−22.14 (3.95)	− 20.61 (3.25)	.31	−18.87 (5.86)	− 16.85 (4.74)	− 16.54 (5.15)	.36	− 19.54 (5.22)	− 18.37 (5.47)	− 17.96 (4.24)	.58
SRs, l/s	−1.42 (0.38)	− 1.44 (0.34)	− 1.31 (0.16)	.61	− 1.24 (0.28)	−1.20 (0.26)	− 1.21 (0.32)	.89	− 1.35 (0.35)	−1.19 (0.37)	− 1.25 (0.21)	.22
SRe, l/s	1.53 (0.39)	1.51 (0.40)	1.27 (0.37)	.056	1.37 (0.37)	1.21 (0.36)	0.96 (0.38)^b^	.04	1.30 (0.43)	1.21 (0.40)	1.29 (0.43)	.75

*Abbreviations*: *EF* ejection fraction, *LV* left ventricular, *RV* right ventricular, *S* strain, *SRe* peak early diastolic strain rate, *SRs* peak systolic strain rate, *T0* baseline (pretreatment), *T1* time from start of chemotherapy to first echocardiogram, *T2* time from start of chemotherapy to second echocardiogram. (SD)

^a^Data are expressed as mean (±SD)

^b^*P* < .05 vs age < 50 years

### Predicting a decrease in LVEF

Combining both RV GLS at T1 and LV GLS was the strongest predictor of cardiotoxicity (area under the curve [AUC], 0.91; sensitivity, 100%; specificity, 73%; *P* < .001). LV GLS at T1 (AUC, 0.85; cutoff, − 14.06; sensitivity, 91%; specificity, 83%; *P* = .003) was also a strong indicator of subsequent cardiotoxicity. Combining LV GLS with longitudinal early diastolic strain rate (LSRe) at T1 (AUC, 0.90; sensitivity, 91%; specificity, 86%; *P* < .001) and combining GLS with radial early diastolic strain rate (RSRe) at T1 (AUC, 0.88; sensitivity, 91%; specificity, 78%; *P* < .001) were also strong predictors of subsequent cardiotoxicity (Table 4).

**Table 4:** ROC Analysis of Echocardiographic Parameters at T1 for Predicting Cardiotoxicity (Decrease of ≥10% to EF ≤53%)

T1 Parameters	AUC	*P* Value	Odds Ratio (95% CI)	Cutoff Value	Sensitivity, %	Specificity, %
GLS, %	0.85	.003	1.47 (1.13-2.04)	−14.06	91	83
LSRs, l/s	0.54	.40	0.40 (0.04-3.46)	−0.86	93	30
LSRe, l/s	0.58	.25	2.55 (0.51-14.28)	0.94	82	47
CS, %	0.57	.04	1.15 (1.01-1.39)	−23.8	96	30
CSRs, l/s	0.58	.24	2.52 (0.55-13.06)	−1.46	67	52
CSRe, l/s	0.50	.98	1.01 (0.30-3.32)	1.0	96	16
RS, %	0.52	.07	0.96 (0.92-1.0)	33.27	56	75
RSRs, l/s	0.54	.65	0.85 (0.4-1.73)	1.86	41	73
RSRe, l/s	0.71	.005	0.33 (0.14-0.73)	−2.66	58	82
GLS + LSRe	0.90	<.001	1.94 (1.36-3.11) 93.40 (3.15-5807.32)	…	91	86
GLS + RSRe	0.88	<.001	1.57 (1.14-2.38) 0.26 (0.08-0.66)	…	91	78
RV GLS	0.53	0.93	0.99 (0.87-1.14)	−14.83	78	44
LV GLS + RV GLS	0.91	<.001	2.43 (1.43-5.34) 0.82 (0.62-1.03)		100	73

*Abbreviations*: *AUC* area under the curve, *CI* confidence interval, *CS* circumferential strain, *CSRe* circumferential peak early diastolic strain rate, *CSRs* circumferential systolic strain rate, *EF* ejection fraction, *GLS* global longitudinal strain, *LS* longitudinal strain, *LSRe* longitudinal peak early diastolic strain rate, *LSRs* longitudinal peak systolic strain rate, *RS* radial strain, *RSRe* radial peak early diastolic strain rate, *RSRs* radial systolic strain rate, *T1* time from start of chemotherapy to first echocardiogram

### Reproducibility

The intraobserver and interobserver agreement are shown in Table 5. Both measurements of agreement were lowest for radial S, SRs, and SRe values.

**Table 5:** Intraobserver and Interobserver Agreement for LV S Parameters

Variable	Intraclass Correlation Coefficient (95% CI)	
	Intraobserver	Interobserver
Longitudinal		
S	0.969 (0.898-0.984)	0.956 (0.905-0.975)
SR	0.872 (0.641-0.944)	0.986 (0.973-0.993)
SRe	0.905 (0.747-0.960)	0.949 (0.875-0.967)
Circumferential		
S	0.957 (0.898-0.984)	0.778 (0.555-0.887)
SR	0.898 (0.754-0.961)	0.997 (0.992-0.999)
SRe	0.719 (0.309-0.892)	0.986 (0.957-0.993)
Radial		
S	0.934 (0.834-0.974)	0.887 (0.627-0.942)
SR	0.866 (0.653-0.946)	0.960 (0.875-0.980)
SRe	0.734 (0.340-0.897)	0.871 (0.477-0.918)

*Abbreviations*: *S* strain, *SR* strain rate, *SRe* peak early diastolic strain rate

## Discussion

This study resulted in several main findings. First, to our knowledge, this is the first study that has shown that combining GLS and RV GLS measurements is a strong predictor of cardiotoxicity in patients with breast cancer who receive anthracycline-trastuzumab treatment. Second, after the first few months of chemotherapy, there was a significant decrease in LV GLS, GCS, SRs, and SRe, even in patients receiving under the upper limit of the recommended cardiac safe dose. Third, we observed significant changes in RV mechanics in our patients, with important decreases in RV GLS, SRs, and SRe at T1 and in S and SRs at T2.

Previous studies have also shown that GLS is the optimal marker for detecting subclinical heart failure (type B) [11, 32]. As a result, we recommend that this measurement be incorporated as a marker of stage B heart failure in this population of patients. Anthracyclines are well known antineoplastic agents that have proven to be cardiotoxic in a dose-dependent manner [8]. For example, doxorubicin has a lower risk of congestive heart failure for doses below 450 mg/m^2^, but it is associated with moderate risk at 550 mg/m^2^ and high risk at more than 1000 mg/m^2^ [29, 33]. Our study population received a mean dose of 466 mg/m^2^, which is below the threshold for moderate risk and slightly above that for low risk. Several studies have shown that when trastuzumab is given alone or combined with anthracyclines it is associated with cardiotoxicity. Previous randomized controlled trials have also shown better survival for patients with HER2-positive breast cancer treated with trastuzumab, a monoclonal antibody that targets the HER2 receptor [15, 24, 34]. For this reason, 2D-STE can help in the detection of subclinical systolic dysfunction in patients given combined chemotherapy, as in our study. These variables, which include S and SR, will allow us to detect early systolic and diastolic dysfunction in order to predict future changes in EF [34, 35].

RV abnormalities may also occur in patients with cancer, although the frequency has not been determined. One study of 37 patients showed a decrease in RV systolic and diastolic indices on echocardiography relatively soon after chemotherapy with anthracyclines; however, most indices were within normal ranges [34]. In our study we observed significant reduction on RV mechanic parameters at both T1 and T2.

Fifty-four of our patients received anthracycline at T1 and anthracycline plus trastuzumab at T2. When compared with the changes from T0 to T1, the differences for T1 to T2 were significant (*P*<. 05 for all), in favor of improvement rather than further worsening of heart failure. These results suggest that patients with breast cancer can better tolerate the appropriate dose of anthracycline-trastuzumab treatment after early changes in myocardial mechanics occur.

### Study limitations and future directions

This study was limited by its retrospective design and small sample size. However, it was able to show that RV mechanical parameters should be studied in a larger population. Our data shows increased risk of cardio toxicity in the older population, however there is not enough power when the data is divided into age groups to draw a definitive conclusion.

## Conclusions

Abnormal values of 2D-STE in the presence of a normal EF can predict a future drop in ejection fraction. GLS should be used as a marker of stage B heart failure in patients given a combined anthracycline-trastuzumab regimen, despite treatment with doses in the moderate-risk range. In addition, S and SR provided data that showed early changes in LV myocardial function (stage B heart failure) after less than 3 months of treatment with chemotherapy.

## Abbreviations

2D-STE: 2-dimensional–speckle tracking echocardiography
AUC: area under curve
EF: ejection fraction
GLS: global longitudinal strain
ICC: intraclass correlation coefficient
IQR: interquartile range
LSRe: longitudinal peak early diastolic strain rate
LSRs: longitudinal peak systolic strain rate
LV: left ventricle
LVEF: left ventricular ejection fraction
ROC: receiver operating characteristic
RSRe: radial early diastolic strain rate
RV: right ventricle
RVLS: right ventricular longitudinal strain
S: strain
SR: strain rate
SRe: early diastolic strain rate
SRs: peak systolic strain rate
